# A network-based cross-sectional study of geographic access disparities to Medicare-participating dermatologists in the United States

**DOI:** 10.1016/j.jdin.2025.09.016

**Published:** 2025-10-10

**Authors:** Clayton Frazier, Congcong Miao, Sara Shalin, Hanna Jensen, Emily Frazier

**Affiliations:** a College of Medicine, University of Arkansas for Medical Sciences, Fayetteville, Arkansas; b Department of Geography, Community, Sustainability, and Urban Studies, University of Connecticut, Storrs, Connecticut; c Department of Dermatology, University of Arkansas for Medical Sciences, Little Rock, Arkansas; d Department of Surgery, University of Arkansas for Medical Sciences, Fayetteville, Arkansas; e School of Earth, Environment, and Sustainability, Missouri State University, Springfield, Missouri

**Keywords:** access, dermatology, disparity, geography, GIS, Medicare, underserved

*To the Editor:* The imbalanced U.S. geographic distribution of dermatologists exacerbates inequities surrounding patients’ access to care, particularly in rural areas.^1^^,^^2^ This national cross-sectional study employs geospatial mapping to investigate patient access to Centers for Medicare and Medicaid Services-participating dermatologists (CMSD). This study offers novel insights into accessibility by employing a 1-h drive service area network (OHSA) around each CMSD’s practice location to clearly define which communities can practically access a dermatologist. This approach addresses limitations in studies exploring accessibility using fixed “as the crow flies” distances from a dermatologist’s clinic or per-capita dermatological density^1^^,^^3^ by providing a pragmatic definition of access to care.

Clinic addresses for 11,831 actively practicing CMSDs were obtained from the 2022 Medicare Physician public database^4^ and mapped in ArcGIS Pro, and OHSAs around each clinic were created using existing road network infrastructures and speed limits. U.S. Census data were overlaid to identify the population within and outside each OHSA. A complete methodology Supplementary Supplement is available via Mendeley at https://data.mendeley.com/datasets/59mv5dfkym/1. Individuals living beyond the OHSA were deemed geographically disadvantaged in accessing a dermatologist.

Fig 1 maps OHSAs for U.S. CMSDs. Of the 331,636,180 people in the U.S., 11,381,025 (3.43%) live beyond the OHSA of any CMSD. There are clear regional distinctions between states in their provision of geographic access to a CMSD. Seven of the 10 states with the smallest percentage of their population living outside any CMSD OHSA are on the densely populated East Coast (NJ, RI, CT, NY, PA, MA, NC), while 8 of the 10 states with the highest percentage of their population outside the OHSA of any CMSD are found in the rural Mid- and Mountain West (WY, MT, ND, SD, NM, NE, IA) and Alaska. These 20 states are listed in Table I.

**Fig 1:**
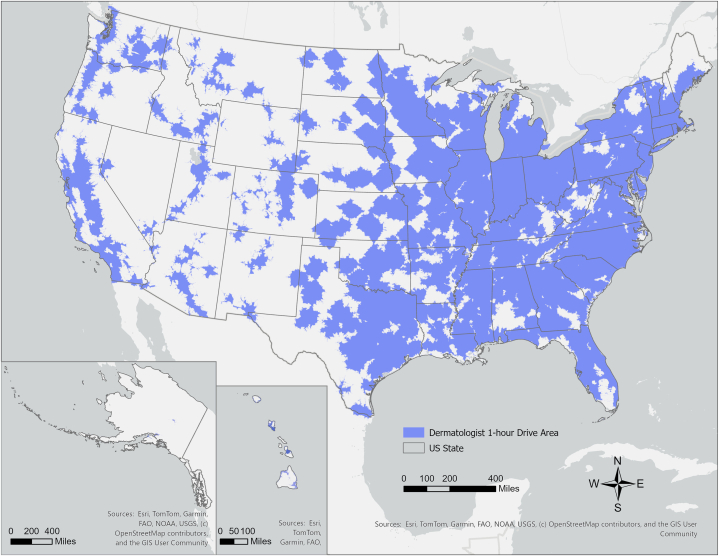
Aggregated OHSAs around U.S. CMSDs.“One-hour drive-time service areas (OHSAs) around all CMS-participating dermatologists in the United States. Areas in white represent geographic gaps in access”. *CMS*, Centers for Medicare and Medicaid Services; *CMSDs*, Centers for Medicare and Medicaid Services-participating dermatologists.

**Table I:** Top 10 U S. states with the highest and lowest percentage of their population outside any OHSA

State	Total population	Population outside any OHSA	Percentage of population outside any OHSA
Top 10 states with highest percentage of population outside any OHSA			
Wyoming	579,761	281,693	48.6%
Alaska	733,971	295,913	40.3%
Montana	1,105,072	323,326	29.3%
New Mexico	2,114,768	573,952	27.1%
North Dakota	779,361	209,830	26.9%
Maine	1,377,400	197,522	14.3%
South Dakota	899,194	126,334	14.1%
Nebraska	1,965,926	257,293	13.1%
Iowa	3,195,937	402,841	12.6%
West Virginia	1,784,462	211,812	11.9%
Top 10 states with lowest percentage of population outside any OHSA			
North Carolina	10,584,340	118,398	1.12%
Massachusetts	6,992,395	64,014	0.915%
Pennsylvania	12,986,518	114,469	0.881%
New York	19,793,038	131,157	0.663%
Illinois	12,692,653	65,018	0.512%
Connecticut	3,598,348	15,656	0.435%
Rhode Island	1,095,371	4492	0.410%
New Jersey	9,267,014	37,348	0.403%
Ohio	11,780,046	26,018	0.221%
Indiana	6,811,752	5967	0.088%
**U.S.A.**	**331,636,180**	**11,381,025**	**3.43%**

*OHSA*, One-hour drive time service area.

Our OHSA approach reveals U.S. dermatologic care deserts. Although regional patterns are associated with access to a CMSD, exceptions exist. Maine, although located on the East coast, has the sixth highest percentage of its population (14.3%) residing beyond a dermatologist’s OHSA, while Mid-Western Indiana offers the greatest access, with just 0.088% of its population living beyond an OHSA. These outliers may arise from road network differences or the presence of academic medical centers.

This study’s novel use of the OHSA reveals although 96.6% of the U.S. population can reasonably access a dermatologist, geographic regions comprising over 11 million people remain underserved. Study limitations exist: other social vulnerability factors modulate geographic access beyond drive-time,^5^ and dermatologists may opt out of CMS participation, both which decrease access. Conversely, teledermatology, satellite clinics, and care provided by nondermatologist providers may increase access.

OHSA-identified care deserts provide advocates in medical education and within state and national specialty societies with a practical roadmap for improving access through rural residency tracks, recruitment and mentoring of rural dermatologists, and incentivizing practicing in OHSA-underserved areas. The OHSA technique can be applied to identify access barriers in other countries and specialties. Future studies should examine how satellite clinics extend dermatologic OHSAs across the U.S. and incorporate social vulnerability factors to holistically define access.

## Conflicts of interest

None disclosed.
